# “We are not hard to reach, but we may find it hard to trust” …. Involving and engaging ‘seldom listened to’ community voices in clinical translational health research: a social innovation approach

**DOI:** 10.1186/s40900-021-00292-z

**Published:** 2021-06-26

**Authors:** Safina Islam, Olivia Joseph, Atiha Chaudry, Davine Forde, Annie Keane, Cassie Wilson, Nasima Begum, Suzanne Parsons, Tracy Grey, Leah Holmes, Bella Starling

**Affiliations:** 1 Public Programmes Team (now Vocal), Manchester University NHS Foundation Trust (currently, Ahmed Iqbal Ullah RACE Centre), Manchester, UK; 2 Public Programmes Team, Manchester University NHS Foundation Trust (currently, Yorkshire Quality and Safety Research Group), Manchester, UK; 3 Manchester BME Network CIC, GM BME Network and Manchester Equalities Hub, Manchester, UK; 4 Manchester BME Network CIC, Manchester, UK; 5 Public Programmes Team, Manchester University NHS Foundation Trust, Manchester, UK; 6 Annana, Manchester Bangladeshi Women’s Organisation, Manchester, UK; 7 Public Programmes Team (now Vocal), Manchester University NHS Foundation Trust, Research & Innovation Division, The Nowgen Centre, 29 Grafton Street, Manchester, M13 9WU UK; 8 NIHR Manchester Biomedical Research Centre, NIHR Manchester Clinical Research Facility, Manchester, UK

**Keywords:** Diversity, Inclusion, BAME, Ethnic groups, Public involvement, Innovation, Community engagement, Translational research

## Abstract

**Background:**

Public involvement in clinical translational research is increasingly recognised as essential for relevant and reliable research. Public involvement must be diverse and inclusive to enable research that has the potential to reach those that stand to benefit from it the most, and thus address issues of health equity. Several recent reports, however, indicate that public involvement is exclusive, including in its interactions with ethnic groups. This paper outlines a novel community-led methodology – a community sandpit – to address the inclusion of ethnic groups in public involvement in research, reports on its evaluation, findings, legacy and impact.

**Methods:**

Through detailed planning – thinking through and taking into account all stakeholders perspectives in the planning and design of the sandpit, relationship-building, co-design and co-delivery between the Public Programmes team based at Manchester University NHS Foundation Trust and the Greater Manchester Black and Minority Ethnic Network - the community sandpit was held in July 2018.

**Results:**

Fifteen community organisations took part in the two-day event, as well as six researchers, and six creative practitioners. Six community-based partnership projects were seed-funded; four of these received additional funding from other sources also.

**Conclusions:**

Evaluation of the sandpit showed the format to be well-received by all: it levelled power relationships between community organisations, health researchers and research infrastructure; it developed capacity amongst researchers about the accessibility, role and potential of community organisations. Described as “not another community seed fund” by community partners, the sandpit offered community partners, equitable avenues for collaboration within Greater Manchester translational research and led to the formation of the Black, Asian and Minority Ethnic Research Advisory Group (BRAG Vocal Website information, - https://www.wearevocal.org/opportunities/black-asian-and-minority-ethnic-research-advisory-group-brag/, 2021). The method has the potential to be replicated elsewhere to support inclusive public involvement in research and inclusive research.

**Supplementary Information:**

The online version contains supplementary material available at 10.1186/s40900-021-00292-z.

## Plain English Summary

Public involvement in “bench to bedside” research (from laboratory-based research to clinical practice) is increasingly recognised as essential for relevant and reliable research. To enable the findings from health research to meet the needs of those who stand to benefit from it the most and to ensure that differences in health and disease are reduced as much as possible, public involvement must be diverse and inclusive. Recent reports, however, indicate that public involvement is exclusive, including in its interactions with racially minoritised groups. This paper outlines a novel community-led methodology – a community sandpit – to address the inclusion of ethnic groups in public involvement in research, reports on its evaluation, findings, legacy and impact.

Through detailed planning – thinking through and taking into account all stakeholders perspectives in the planning and design of the sandpit, relationship-building, co-design and co-delivery between the Public Programmes Team (now Vocal) based at Manchester University NHS Foundation Trust worked with the Greater Manchester Black and Minority Ethnic Network - the community sandpit was held in July 2018. Fifteen community organisations took part in the two-day event, as well as six researchers, and six creative practitioners (artists with experience of working with the public on socially engaged projects to engage them in areas such as science). Six community-based partnership projects were seed-funded; four of these received additional funding from other sources. Evaluation of the sandpit showed the format to be well-received by all: it levelled power relationships between community organisations, health researchers and research infrastructure; it increased researchers knowledge and insights about the accessibility of community organisations and of how they might work effectively with them. Described as “not another community seed fund” by community partners, the sandpit began the process of levelling the playing field for collaboration between Greater Manchester translational research and local community organisations and led to the formation of the Black, Asian and Minority Ethnic Research Advisory Group (BRAG) (Vocal Website information, - https://www.wearevocal.org/opportunities/black-asian-and-minority-ethnic-research-advisory-group-brag/, 2021). The method has the potential to be repeated elsewhere to support inclusive public involvement in research and inclusive research.

**Supplementary Information:**

The online version contains supplementary material available at 10.1186/s40900-021-00292-z.

## Background

### Diversity in public involvement in health research in the UK

Public involvement is an increasingly accepted component of ethical and relevant health research in England, including through one of its major funders: the National Institute of Health Research (NIHR) [1]. A strategic review of public involvement in England, however, highlights the need for public involvement to become more diverse and inclusive:

*“A diverse and inclusive public involvement community is essential if research is relevant to population needs and provides better health outcomes for all. We have been struck by the degree to which researchers and public contributors have encountered barriers when trying to work with different communities and populations*.” (Appendix four – Going the Extra Mile Recommendations) [2]

A recent survey highlights that the majority of public contributors to NIHR research are older (62% 50–79- UK-34.8%), female (58%; UK-50.6%) and White British (77% - UK-87.2%) [3]. Young people and minority ethnic communities are under-represented in NIHR public involvement. Only 2% of public contributors surveyed in 2018–2019 were under 25 (UK – 18.9); 14% are aged 26–49 (UK population. Asian ethnic groups represent only 3% (UK-4%), Black ethnic groups only 2% of NIHR public contributors (UK-3%). Research carried out by the Health Research Authority shows that people from ethnic and lower socioeconomic groups feel far less confident about being treated with dignity and respect in research compared to their White and higher socioeconomic counterparts (35% versus 50%) [4]. Likewise, in the related field of science communication, informal science education has been ‘exclusive’, and many audiences are ‘underserved’ by science communication (cf. British Science Association audience map) [5, 6].

### Barriers to inclusion of ethnic communities in research

A growing evidence base explores reasons for the low levels of public involvement of ethnic groups, including Black, Asian and Minority Ethnic (so called “BAME”) groups in clinical and translational health research [7]. Analyses of research participation in cancer clinical trials, mainly from the US [8–10] with some from the UK [11, 12] and trials related to other conditions such as asthma [13] and mental health [14] in South Asian and African-Caribbean communities in the UK and abroad, provide complementary evidence for barriers to involvement. Strategies to reduce inequalities in healthcare have identified the representation of diverse groups in clinical research as an important component of research studies [15, 16].

Participation, engagement and involvement in health research are different but related areas [17]. All can be adversely affected by a lack of diversity in those participating in and becoming engaged and involved with health research. Using both the existing literature and our collective experience of engaging and involving diverse audiences with scientific research, we have summarized barriers to inclusion of diverse communities in health research across participation, engagement and involvement (Table 1). Where possible we have indicated where barriers have been referenced within the literature and were barriers were reflections from our practice.

**Table 1:** Barriers related to participation, engagement and involvement of ethnic groups in health research

**Barriers related to research culture**	
Attitudinal barriers	Researchers (along with the rest of society) may have unconscious bias or preconceptions (stereotypes or cultural myths) about whether patients from certain groups are interested in participating in a study or in research in general (Practice observation)
Low awareness of the importance of inclusive research and diverse recruitment	The historical and conceptual understanding of race, ethnicity and culture can make the recruitment of people from ethnic minority backgrounds appear more problematic to researchers (Practice observation)
Study design	Study design (including development of inclusion and exclusion criteria) may structurally exclude BAME patients and those with lower socioeconomic status, as they tend to have poorer health in general. (Practice observation) Overly complex, jargon filled, study information and consent forms can exclude potential participants from different backgrounds even when their command of English is good.
Language, communication and cultural barriers	Often there is no guidance or resources for researchers to help them include patients who do not speak English as a first language, meaning these patients are excluded from a study. Ethics committee requirements to translate all written material in different languages can have little positive impact when many community languages are not generally used in a written form, or low levels of health literacy are not accounted for in the material. (Practice observation)
Increased cost of studies	The perception that the addition of extra variables such as ethnic diversity of participants would increase the cost and duration of research studies, through the requirement of more sub-group analyses and increased recruitment costs such as translators (Practice observation)
**Barriers related to healthcare**	
Structural health inequalities and racism	So-called ‘underserved’ populations can expect higher incidence rates of conditions (eg. Cancer) because of structural exclusion and unmet need (eg. Inadequate access to prevention and screening perhaps due to poor information on prevention and / or screening appointments being held at times and in locations which are inaccessible for particular groups, later diagnosis, exclusion criteria/recruitment bias in research studies) or and/or unmet need through exclusive research priorities and design.
Power dynamics	Power dynamics inherent in social and health inequalities acknowledge that the structure and models for involvement (e.g. use of meeting rooms, rigid agendas, chairing of meetings) can be exclusive or culturally imperialist, ie. Where the dominant research culture significantly affects how research or public involvement is conducted. Is this conducive to the development of trust, valued involvement and creation of equal knowledge spaces? [18–20]
**Barriers related to society and heritage**	
Mistrust of anchor institutions and/or healthcare providers	Often cited as the most common barrier to the participation of ethnic groups in clinical trials. Poor previous experiences and low satisfaction in a healthcare or other institutional can lead to people from ethnic and lower socio-economic groups in research feeling less confident about being treated with dignity and respect in research Distrust around sharing or misuse of personal information and data protection issues is also higher in ethnic groups (cf. [4])
**Barriers within communities**	
Lack of understanding of the research process	In disadvantaged and marginalised communities, this can lead to a rejection to an invitation to participate. (Practice observation)
Socioeconomic status	Decisions by patients to participate may be driven in part by socio-economic status. Loss of income (actual or perceived) or costs incurred by participation, engagement and/or involvement in research (due to increased hospital visits, for example) may deter participation. (Practice observation)
Flexibility	Lack of flexibility around timing can prevent many patients and carers from participation, engagement and/or involvement. Common reasons include childcare, carer responsibilities and employment in sectors that wouldn’t approve extra time off to attend participation or involvement activities. (Practice observation)
Stigma	Different cultural and or religious beliefs of patients may impact upon their perceptions of health, research and participation in a clinical trial. (Practice observation)

### Working with seldom listened to communities in a social innovation approach

Some helpful guides to working with racially minoritised groups and communities in research already exist [21–24] and have been published during the preparation of this paper [25]. To address greater inclusion, and explore some of the deeper barriers to engagement and involvement in health research, beyond tokenism or a temporary solution, we piloted a new approach – a community sandpit – based on principles and practices of social innovation and co-production [26].

Sandpits are intensively facilitated workshops, which aim to uncover innovative solutions. They bring together a mix of people, give them time to talk and exchange ideas, and fund the projects that they come up with. We chose this model to encourage community-led innovation in public involvement because it allows different sectors to recognise the knowledge and expertise that they can bring and provides a safe space to experiment and find collective solutions. It’s also a fun and engaging approach and uses creative activities and tools, which enable new dialogue and ideas to emerge.

We have previously reported on our strategic approach to working across public engagement and involvement, [17, 27] which includes a focus on diversifying the audiences and producers of public involvement and effecting a shift from ‘research led’ engagement, involvement and research to ‘community-led’ engagement, involvement and research. The Public Programmes Team (now Vocal) leads the Patient and Public Involvement and Engagement (PPIE) Strategy for the NIHR Manchester Biomedical Research Centre (MBRC) and the NIHR Manchester Clinical Research Facility (MCRF) and the NIHR Manchester Patient Safety Translational Research Centre (GMPSTRC). These NIHR infrastructure have strategic objectives to deliver effective and innovative (PPIE) and to increase the diversity of people involved in shaping research. Therefore, a key starting point was to build relationships with community organisations and explore with them how having a voice in health research may be relevant and useful for people in their communities. Here we report on the methodology and impact of the sandpit, and suggest it as a replicable way to work with communities in community engagement and involvement to ultimately foster inclusive research. We also report on the legacy of the sandpit.

## Methods

**Fig. 1:**
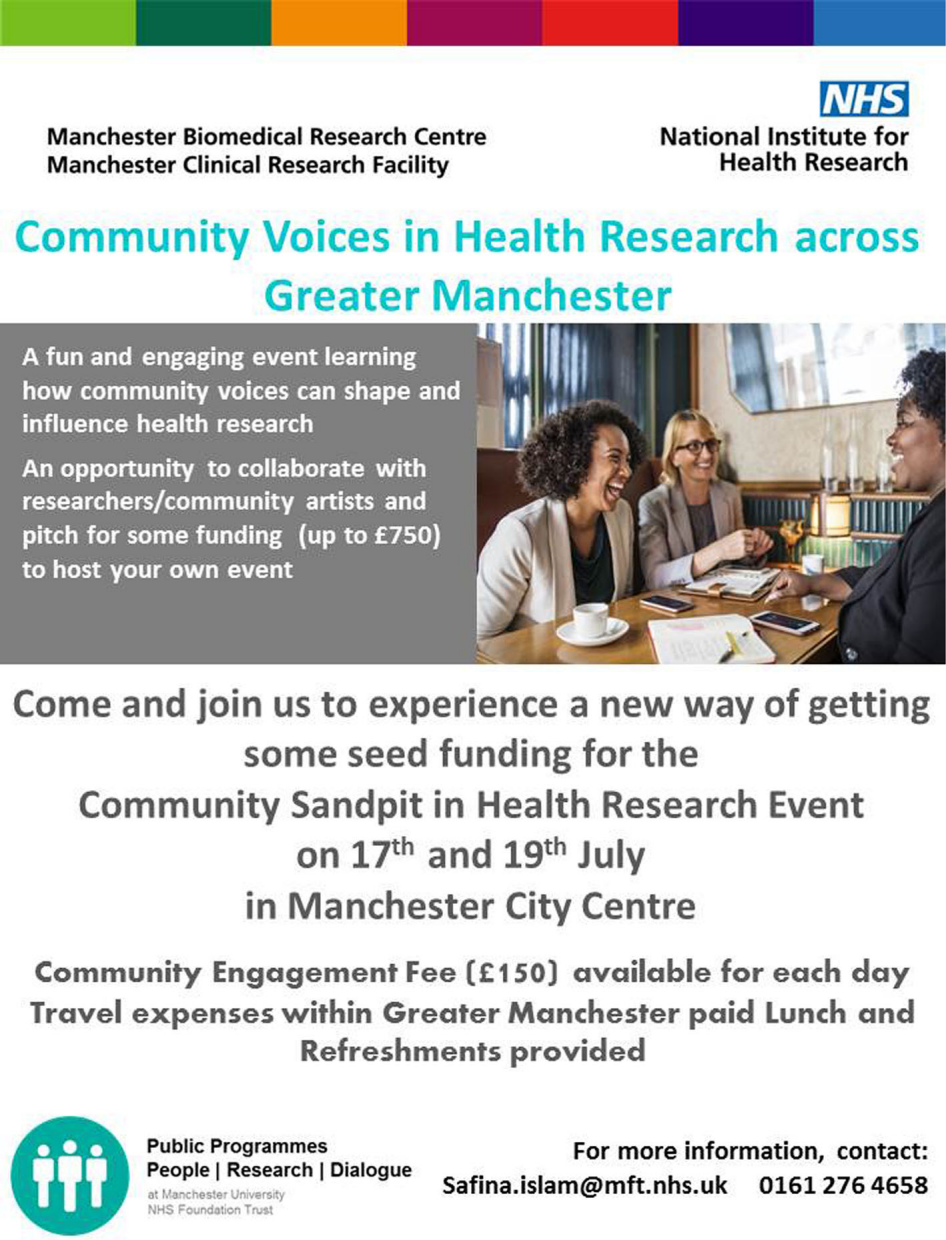
Event flyer

In July 2018, a two-day community sandpit event brought together over 30 Voluntary and Community Sector (VCS) organisation representatives, creative practitioners (artists with experience of working with the public to engage them in areas such as science)., community leaders, researchers and public involvement practitioners from across Greater Manchester (Fig. 1, event flyer).

### Aims and principles

The aims for the sandpit were to:

- Build meaningful relationships with diverse community organisations and health research
- Administer a community seed fund to explore community led innovation in the sector
- Facilitate and fund community-led innovation
- Level the playing field between community organisations and researcher anchor institutions by bringing researchers, community organisations and artists together to collaborate and explore solutions for the lack of diversity of people involved/participating in health research

At the outset, we elaborated some key principles to apply to the sandpit. We:

- Emphasized a co-design approach when working with community partners
- Valued community organisations as experts rather than gate keepers or community leaders
- Viewed community organisations, researchers and creative practitioners all as experts
- Put health research in contexts that have a practical interest to community groups and their users
- Used creative approaches to trigger discussion and debate and create a learning together environment
- Valued the time and expertise of community organisations
- Used methods to level the playing field between different types of community organisations
- Enable community organisations to develop their own ideas and pitch for funding but avoid a typical ‘seed funding’ process where organisations have to invest their own time and resources in developing the idea

### What we did

#### Sandpit partner

Through existing voluntary and community sector (VCS) contacts, the Public Programmes Team (now Vocal) [28] initially mooted the idea of a sandpit, and then sought active and equitable partnership with the Greater Manchester Black and Minority Ethnic Network (GMBME) [29] to develop the idea further, then co-design and promote the Community Sandpit event. The GMBME has extensive knowledge of the VCS, wide reach amongst grassroots community organisations, as well as having supported different health promotion and infrastructure development initiatives across GM.

The partnership between the Public Programmes Team and GMBME was key to this initiative; we were equal partners in the development of the project and established a clear framework and agreement for our roles, responsibilities and commitments. This partnership based on mutual goals meant that the event was designed with a real understanding of what community organisations would value. There was a higher demand for places at the event than we had originally expected. The event was designed to ensure that organisations that wanted to take part did not have to fund their own staff time.

#### Relationship development, co-design, promotion and recruitment

Active involvement in health research was a new focus for many of the community organisations who would eventually take part in the sandpit. Working with Black, Asian and ethnically diverse community groups was also a new area for many of the researchers taking part. A significant amount of time was spent developing relationships both with researchers and with GMBME members and community organisations, to give background to the initiative, answer questions and secure buy-in. As an established community organizer and enabler SI was already a trusted individual within grass roots community groups in GM. The Public Programmes team is a trusted partner for researchers in GM, working closely with researchers from NIHR research infrastructure to lead their public involvement and engagement work and so already had some pre-established relationships that facilitated researcher buy-in.

Working in partnership between the Public Programmes Team and the GMBME Network, the event was designed to ensure that organisations putting forward staff or volunteers to participate did not have to resource staff time to attend. Staff/volunteer time was valued at the rate NIHR INVOLVE recommends for patient contributors and payment made to organisations or individuals as appropriate [30]. NIHR INVOLVE was the umbrella organization for patient and public involvement in NHS research, which provided support to patients and the public wanting to become involved in research, and to researchers wanting to involve people; it has now become the NIHR Centre for Engagement and Dissemination [31].

The event was designed to enable participants to explore context and practice of PPIE and health research in relation to their own interests and communities. Discussion about how PPIE could be improved and how participants could have a role in transforming the sector were also incorporated into the event design. The GMBME network used its existing communication channels that included the web presence of its parent organization, GM Centre for Voluntary Organisation (GMCVO), one of the largest VCS infrastructure support organisations in the GM conurbation, its own email newsletter mailing list and network events and meetings leading up to the registration deadline for the event to promote and recruit staff. A promotional flyer was also produced (Box 1).

#### Sandpit event

The community sandpit were delivered over 2 days with a one-day break in between to allow participants time to process what they had learnt and think about the second day.

Day one consisted of facilitated activities to enable representatives from community organisations/artists to familiarise themselves with public involvement and health research in the GM context, including the activities of the Public Programmes Team, and NIHR research infrastructure in GM: MBRC, MCRF and the GM PSTRC. The first day was highly interactive with lots of opportunity for discussion and debate, including activities designed to address different power dynamics in the room.

The activities included “the unseen” (a creative activity enabling participants to experience the process of discovery and their own reflexivity in developing research questions) and role-play where participants experienced a mini-patient research panel with some actual research examples.

On day two, researchers joined the discussions with community organisations and artists, there were presentations relating to health conditions and prevalence in diverse communities, followed by speed networking where researchers and artists presented what specific skills they could add to any projects pitched. The participants then worked in small groups to devise a new project/event that would engage their communities in health research, with a view to developing a research question and/or project of relevance to communities. Whilst designing their projects they were able to call on researchers and artists to be included as project partners if they felt them to be a good fit. Each project was pitched to the whole audience and after an interactive audience voting process (which included all sandpit participants and facilitators) the winning projects were awarded funding (between £500–£750). All sandpit participants (community organisations, researchers, artists and members of the Public Programmes Team) were able to vote for projects. Having heard the project pitches everyone was asked to write down their top three preferences. Preference one was given three points; preference two, two points and preference three, one point. The points achieved by each project were then added up and the top five scored projects received funding.

### Evaluation

#### Evaluation against sandpit aims

We considered the following aspects within the evaluation process:

- Practical consideration of the actual event and recruiting to the event
- Researcher/ practitioner/ participant and partner perspectives and experience

#### Evaluation methods

The evaluation methods used included

- A survey of sandpit participants
- Interviews with researchers and a focus group of community organization representatives
- Observations and reflections of the event and recording the topics discussed

At the end of each day of the sandpit, a simple questionnaire with 10 questions captured a mixture of quantitative and qualitative indicators against the sandpit aims. For each day a different questionnaire was used which focused on the specific activities on that day. Questions focused on how well the participants felt the activities went, whether they were able to contribute and whether they felt listened to, and space for some free text responses. The questionnaire was sent to all sandpit participants on each day (day one *n* = 21; day two *n* = 27).

Frequencies were calculated for each questionnaire response and free text data was, where possible, organized into themes. Just the evaluation questionnaire data is presented within this paper.

#### Follow up

Where required, staff from the GMBME Network and/or the Public Programmes Team supported the community organisations that won pitches to deliver their projects and record/observe their community reach. This happened through project delivery, supporting or refining the scope of the project, and its evaluation. An informal feedback and sharing event was held in Feb 2019 where funded community organisations were invited to share the outputs and any learning from their projects with the whole group.

## Results

### Summary of the sandpit (Table 2)

**Table 2:** Community sandpit at a glance

**Expressions of interest from community organizations**	30
**Number of community organisations selected to participate**	15, selected by the Public Programmes team and GMBME, to ensure a broad representation of different communities of interest, geographical spread across Greater Manchester and experience of working on health issues
**Number of creative practitioners taking part**	Six, including digital, visual, audio and spoken word media.
**Number of researchers taking part**	Six, from the MBRC, MCRF, GM PSTRC, University of Manchester
**Ideas for projects**	14
**Pitches funded**	Six (totaling £3100)
**Projects subsequently receiving additional funding**	Four (including through Health and Primary Care commissioning and public engagement funding)

### Funded projects

The following projects won pitch funding and were delivered between September 2018 and January 2019 (Table 3).

**Table 3:** Community-led research and engagement projects funded through the Community Sandpit

Project name	Project description	Project partners
Skin deep	Focused on skin problems and treatments in the African community in South Manchester. The project aims to engage up to 50 parents and children, and to help researchers identify key research priority for African skin health.	AfroTots and researchers in the Manchester BRC Dermatology theme
Shisha or no shisha	An action research project capturing responses from people who frequently visit Wilmslow Road area where most of the City’s Shisha Cafes operate. A 5–10 min film of the project will indirectly raise awareness about the effects of Shisha smoking and stimulate insights into people’s attitudes towards Shisha smoking.	A partnership between the Ethnic Health Forum, C4Change, GMBME Network, University of Manchester and the Public Health Team at Manchester City Council
Amplifying Voices	A training toolkit to enable authentic ethnic voices to be amplified within health research, across GM, to support research to become more culturally appropriate and influence health outcomes.	A partnership with Afro Tots, Dynamic Support, CS-UK, the New Testament Church, Alchemy Arts, Ethnic Health Forum, Wonderfully Made Woman, and Big People’s Music.
Speak out your voice is your power	A community leaders engagement workshop focused on domestic violence for Black Nigerian women during Black History Month in Central and East Manchester. The project aims to support researchers to understand how some isolated and vulnerable women can be engaged in research on domestic violence.	Wonderfully Made Woman
Menopause monologues	The project will provide opportunities for up to 20 South Asian women to talk about their experiences and tell stories about menopause – a taboo and sensitive subject in some South Asian cultures. Stories will be recorded to engage further audiences in a peer-to-peer approach. The project aims to empower women to take an active role in health and research related to the menopause; to stimulate research into South Asian women’s experiences of menopause; to help shape health care services regarding menopause; to break down barriers to engagement; to promote access to menopause services for South Asian women; to breakdown menopause myths, stigma and taboo	Community artist
Leading through lived experience for positive change	The project aims to share good practice of how lived experience leaders have set up initiatives and projects to improve health and well-being outcomes for people who access their project or initiatives and to learn how the lived experience leaders expertise can be utilised to support community engagement with research	Shining Stars community group

### Evaluation findings

An evaluation questionnaire was completed by sandpit participants at the end of each day. 16/21 participants replied to the questionnaire on day one and 20/27 participants replied to the questionnaire on day two (There were more participants on day two as researchers also attended on this day). Five researchers were interviewed, and 12 community organization representatives took part in a focus group.

### Sandpit format

Data is presented from the sandpit evaluation questionnaires in Tables 4 and 5.

**Table 4:** Quantitative responses to evaluation questionnaire day one *N*=16

	**Yes**	**No**	**Sometimes**	**Non applicable**	**Non applicable**
**Were you happy with the communication until the event?**	15/16	1/16	0/16	N/A	N/A
**Was there anything else you would have liked to have known before coming to the meeting?**	4/16	12/16	N/A	N/A	N/A
	**Completely**	**Partially**	**Not at all**	**I didn’t raise any questions**	**Non applicable**
**If yes, where your questions answered?**	11/16	1/16	0/16	3/16	N/A
	**Yes**	**No**	**Sometimes**	**Non applicable**	**Non applicable**
**Was there enough opportunity to ask questions?**	15/16	0/16	1/16	N/A	N/A
**Did you feel your views were valued?**	16/16	0/16	0/16	N/A	N/A
**What did you think of the activities you were asked to participate in?**					
	**Very**	**A little**	**Not very**	**Not at all**	**Not applicable**
**The unseen**					
It was interesting	13/16	3/16	0/16	0/16	N/A
It was clearly explained	11/16	5/16	0/16	5/16	N/A
I felt able to contribute	14/16	2/16	0/16	0/16	N/A
**The mini patient panel one**					
It was interesting	16/16	0/16	0/16	0/16	N/A
It was clearly explained	14/16	2/16	0/16	0/16	N/A
I felt able to contribute	16/16	0/16	0/16	0/16	N/A
**The mini patient panel two**					
It was interesting	14/16	2/16	0/16	0/16	N/A
It was clearly explained	15/16	1/16	0/16	0/16	N/A
I felt able to contribute	15/16	1/16	0/16	0/16	N/A

**Table 5:** Quantitative responses to evaluation questionnaire day two *N*=20

	**Yes**	**No**	**Sometimes**	**Non applicable**	**Non applicable**
**Was there enough opportunity to ask questions during the meeting?**	20/20	0/20	0/20	N/A	N/A
**Did you feel that your views were valued?**	19/20	0/20	1/20	N/A	N/A
**What did you think about the activities you were asked to participate in?**					
**The speed meeting**					
	**Very**	**A little**	**Not very**	**Not at all**	**Non-applicable**
It was a good way of networking	16/20	2/20	1/20	0/20	N/A
It was clearly explained	17/20	2/20	1/20	0/20	N/A
I felt able to contribute	18/20	1/20	1/20	0/20	N/A
	**Very**	**A little**	**Not very**	**Not at all**	**Non-applicable**
**The project pitch**					N/A
It was fun and engaging	19/20	1/20	0/20	0/20	N/A
It was clearly explained	17/20	2/20	0/20	1/20	N/A
I felt able to contribute	19/20	1/20	0/20	0/20	
	**Very**	**A little**	**Not very**	**Not at all**	**Non-applicable**
**Voting for projects**					
It was a good way of quickly choosing winners	16/20	4/20	0/20	0/20	N/A
It was clearly explained	16/20	4/20	0/20	0/20	N/A
I felt able to contribute	18/20	2/20	0/20	0/20	N/A

The format of the sandpit was felt to be engaging and relevant.

*“Very clear and transparent event”* (Participant 8)

*“I hope we will get to do another session. It was an eye opener.”* (Participant 9)

“*It was fun and relaxed but with a really focused and important message*” (Participant 14)

Within the day one evaluation questionnaire, of the 16 people who responded, 16/21 felt they were adequately prepared for the event, answering ‘No’ to the question: *Was there anything else you would have liked to have known before coming to the Sandpit?* A few expressed suggestions for improvement – they would want to have.

*“a better/clear understanding of the project overall,* i.e. *purpose of pitch on day 2”* (Participant 3)

*“a programme of the day’s activities, or some case studies of projects that have come out of previous sandpits so I knew a bit more about the process before attending”* (Participant 11)

Amongst questionnaire respondents, the activities were felt to be ‘interesting’ (The Unseen – 13/16; Mini patient panel one – 16/16; Mini Patient Panel two – 14/16. 16/20 found the speed meeting a good way of networking (Table 4).

19/20 respondents found the pitch process ‘fun and engaging’. The voting process was found to be a good way of choosing winning projects quickly, by 16/20 of respondents and 18/20 felt able to contribute to choosing winners (Table 5). Two community groups decided not to pitch due to having some reservations: some felt that the funding pots were not enough to scope or pilot a meaningful project and some felt they needed additional time to develop a pitch. Four of the community participants felt strongly that only community partners should have a vote.

*“It did feel that those who knew each other voted for each other”* (Participant 3)

### Was the sandpit inclusive? Did it ‘level the playing field’?

By inclusive we meant that all sandpit participants felt they could contribute equally to the sandpit process and outcome, that their views and voices were heard and that they were positive that they could work collaboratively and effectively with participants from other backgrounds. Data from the evaluation questionnaires suggested that attendees found the event inclusive:

- 16/20 felt that their ‘views were valued (after Day one)’
- 100% felt that ‘the sandpit a good way of getting people to work together (after day two)’
- 18/20 felt able to contribute

Participants – community organisations, artists, researchers and facilitators – felt that the event had addressed power dynamics between researchers and communities, enabling a ‘safe space’ and more level ‘playing field’:

*“First-time in a long time a piece of real collaborative work was done, where we felt really valued”* (Participant 1)

*“We talk about collaboration and partnership working but it doesn’t actually happen. This event put community organisations on an equal footing and values their time and input, even between community organisations”* (Participant 2)

*“It worked really well and allowed people with a safe space to work together”* (Participant 4)

*“Everything worked really well. The researchers were amazing and willing to be challenged”* (Participant 13)

People learned about each other and about the work of their organisations.

*“I learnt so much about the many diverse activities and the work of the organisations that support them”* (Participant 14)

*“It was a day for me to learn something that I didn’t know before. It has increased my knowledge about health research.”* (Participant 6)

### Addressing inclusion in health research and involvement

Much of the discussion and dialogue throughout the 2 days focused on the lack of diverse voices being involved in health research. There were different levels of understanding as to the impact of this at the beginning. By the end of the 2 days increased awareness of this issue evolved, and a call to action for health research and involvement to work more in partnership with community organisations, including with community artists emerged.

“*Yes - a really brilliant first step in getting researchers, NHS, Academics talking to artists for the betterment of our community and to have these community voices heard*.” (Participant 5)

*“I thought it was an excellent opportunity for us to get together to find out more about each other and to network and plan projects together.”* (Participant 3)

*“It was a brilliant way of getting to know and working with others that I do not usually get to work with or did not know prior to this event.”* (Participant 13)

Some participants talked about the rhetoric and language around so-called ‘hard to reach groups’ in health research and involvement:

*“We are not that hard to reach, but we might find it hard to trust you …*”

(Participant 1)

*“As a researcher, really opened my eyes to the fact that engaging communities wasn’t that hard. We are told almost from the start as students that they are hard to reach.”*(Participant 17)

### Reflections from researchers

Overall, researchers found the day valuable and it challenged some of their conceptions of community engagement and public involvement in research:

“*Maybe there should be ‘how to involve researchers in communities’ rather than involving communities in research?”* (Participant 2)

The majority of researchers were happy to work with community participants as part of the sandpit, to develop their ideas and design projects around health research and involvement. A minority (one researcher) would have preferred the sandpit participants to focus on specific ways to engage communities in their existing research projects and that researchers should have attended both days. Another felt that the sandpit wasn’t linked enough to research and that attendance from more researchers (junior and senior) would have been beneficial.

*“Not much link to research. Few/no pitches mentioned involving researchers - mostly about health promotion. Attendance and contribution from more researchers and more senior researchers”* (Participant 2)

### Unexpected outcomes- sandpit legacy

As well as following through the funded projects, the legacy of the sandpit includes:

- The GMBME network as an established ‘go-to’ partner of the Public Programmes Team. We continue to partner up on projects as appropriate and to mutually signpost to issues of common interest.
- The establishment of a BAME Research Advisory Group (BRAG) [32] as part of the Public Programmes Team and the MBRC and MCRF.
- Three community organisations are now directly involved with MBRC led-health research projects/project scoping by providing expertise and community knowledge
- One community participant from the Sandpit has now joined an internal MBRC and MCRF strategic group addressing health inequalities in its work, as a public contributor [33].
- Many participants reported that they were more confident in engaging with health research, 4 of the 16 groups applied to the British Science Week Community Grants Scheme to deliver and science engagement project. Something that we felt that they would not have applied for without having attended the sandpit.

## Discussion: what did we learn?

### How did we do against our aims?

Overall, we feel that we successfully addressed our aims of:

- Building meaningful relationships with diverse community organisations and health research
- Levelling the playing field between community organisations and researcher anchor institutions
- Administering a community seed fund to explore community led innovation in the sector
- Bringing researchers, community organisations and artists together to collaborate and explore solutions for the lack of diversity of people involved/participating in health research

### Format of the sandpit

The following comes from our observations and reflections on the sandpit and the questions and comments made by attendees on the day and from the evaluation questionnaire on each day. Evaluation questionnaire data suggests that the sandpit format was accessible, engaging and inclusive for all involved. Getting ‘hands-on’ including through creative activities and, for example, mock ‘patient panel’ exercises helped to bring to life both the sandpit environment and the reality of what public involvement can ‘look like’. In line with our published approach [17] creative approaches triggered discussion and debate which led to rich and meaningful dialogue. Having presentations about health and research contexts related to GM and to the community organizations present, also helped to make the context of the sandpit relevant. Making content relevant to the audience supported more long term thinking about what needs to happen in the sector and how participants could have a role in its transformation.

It was important for us to create a ‘safe space’ at the beginning of the sandpit. By a ‘safe space’ we mean creating an environments where people can be confident that they are valued, will be listed to and won’t be exposed to discrimination, criticism, harassment or emotional or physical harm. The event was purposefully located (in a neutral space in Manchester Central library), designed and promoted without much information about the format and content, to enable everyone to start at the same ‘place’. Whilst this approach was welcomed by the majority of the participants, some were uncomfortable about not knowing what was expected of them and would have liked much more time to prepare (Evaluation questionnaire). Overall, however, our evaluation shows that the sandpit format enabled a safe, respectful and equal exchange of ideas and skills.

To subvert some of the usual power relationships [18–20] in public involvement in health research where the content, approach and format of public involvement can often be led by researchers, Day one of the sandpit did not include researchers, in order to provide background, context and help create the ‘safe space’. A minority of researchers questioned this approach (Evaluation questionnaire). However, in the preparation of the sandpit, when researchers were canvassed about attending an event, many would have been unable to come away from the demands of their research schedule for two full days. This is relevant to a wider discussion about how much time and value research culture accords to public involvement.

Overall, the ‘pitching’ process was successful. Some participants felt that they would have liked to have more time to prepare their pitch possibly starting on the first day (Evaluation questionnaire). Two community groups decided not to pitch. This was because they felt that the funding pots were not enough to scope or pilot a meaningful project and some felt they needed additional time to develop a pitch. Whilst we had co-designed what we thought was a fair voting process, at least four community participants felt strongly that researchers and/or members of the Public Programmes Team should not have a vote (Evaluation questionnaire). They felt that potentially, different projects would have been funded if only community participants had a vote and this would give an indication of what matters more to communities regarding health research. This is something to bear in mind for future sandpit design.

We learned to be prepared for unexpected interpretations of the pitch ‘brief’ despite time being allowed as part of the sandpit design for participants to think about the pitch brief (in this case – designing a project that would encourage their service users/communities to get involved in health research) a few pitched projects focused more on more traditional health promotion (Evaluation questionnaire). Whilst this could be considered a ‘failure’ of the process, it enabled further group exploration of the health issues at hand, and their relation to research. Taking the time to engage with these ‘health promotion’ pitches also allowed further trust and dialogue to be established with community partners.

### Co-design/partnership process

We invested time to try to ensure all partners felt that they were equal in the development of the project whilst working with a clear understanding of our roles and responsibilities around delivery. The time needed to develop trust and build relationships with community partners needs to be built into the development phase of a co-designed project of this nature [22]. A positive partnership was created between the main partners (the Public Programmes Team and the GMBME Network) which was then extended to the community groups that make up the network and the researchers associated with the Public Programmes Team. This resulted in a much higher than expected response from groups wanting to participate in the sandpit event.

Additional benefits of working with a network as a community partner included being able to use existing communications channels, having access to local intelligence about what health topics were already an issue in different communities and what other initiatives were currently happening in different geographical areas that would prevent (or incentivise) certain groups from engaging with this project.

### Conscious, asset-based community engagement

Our starting point was to focus on building relationships with community organisations as experts in reaching their communities, having existing trust and relationships with individuals who attend their centres and groups. Using this asset-based approach was valuable. Rather than seeing community organisations as ‘gatekeepers’ or conduits to reach individuals or service users in their communities, trust was established by valuing the assets of community organisations in their own right. Creative practitioners were similarly valued in the context of the increasing evidence that arts-based approaches can positively address social and health issues [34, 35].

We tried not to use a traditional ‘seed funding’ process where community organisations are required to invest energy and resources to compete for a small amount of funding (that they might not get). Even when successful, traditional seed funding does not always cover the costs of upfront investment let alone project delivery. Therefore our approach was designed to value community organisations time and expertise. Community participants was valued through payment to their organisations in line with NIHR INVOLVE guidance and in the time spent by the project team before the event and after the event liaising with project partners and funded projects [30]. Participants from smaller grassroots organisations reported that they felt they were better placed to interact with the larger voluntary sector organisations. This was considered a benefit as larger voluntary sector organisations are normally better placed to access small pots of funding because they have often have access to additional resources, such as a fundraising officer as all the pitching and voting was done within the 2 days of the community sandpit.

### Addressing inclusion in public involvement in health research

The funded projects all addressed issues of diversity and inclusion in health research. They have reached deeper into communities to engage and involve diverse audiences. At least one of the funded projects has also led to a grant application for research, with the community partner as a co-applicant. Several of the other projects have gone on to receive further funding. It appears from the sandpit and from our observations and evaluation data that the process and the projects have increased the capacity and capabilities of community organisations to take an active role as partners in both health research and public engagement with health research.

Projects that were funded were quite ambitious and this required some support to get them on track and feasible within the time and resources. If the sandpit is repeated, adequate resources need to be built in to support project delivery and evaluation.

Participants valued the time getting to know each other’s ‘worlds’. Researchers appreciated understanding more about community organisations (including community artists), how they work, and who they work with; community organizations felt able to understand more about research and research culture (Evaluation questionnaire). Of particular interest, the comments made about the language of ‘hard to reach’ revealed conflicting perspectives, but ones that might have become closer through the sandpit activities:

*“We are not that hard to reach, but we might find it hard to trust you …”*

*“As a researcher, really opened my eyes to the fact that engaging communities wasn’t that hard. We are told almost from the start as students that they are hard to reach.”*

The sandpit was felt to ‘open researchers’ eyes’ about working with ethnic communities partnering and valuing community organsations. It highlighted the diversity of community organisations to researchers, how grassroots organisations work and the intricacies of their relationships both within communities and with health and research providers. In addition, some of the comments from researchers reveal the prevailing research culture as viewing public involvement as an intervention rather than a relationship. For example, the expressed desire for the sandpit to focus on engagement with existing research projects focused on a transactional, instrumental view of public involvement rather than a research culture that values the time taken to establish trust between partners. Although we appreciate that there may be room for both models of public involvement often in the same project.

The unexpected outcomes of the sandpit, notably the establishment of the Black Asian and Minority Ethnic Research Advisory Group (BRAG) [32] and the membership of the MBRC and MCRF Health Inequalities Steering Group [36], have the potential to strategically influence research culture to ensure more inclusive practices.

The evaluation of the community sandpit suggests that there was enthusiasm and energy to continue to foster an engaged relationship between community organisations and health research. Eight community connectors expressed an interest and two meetings were held to discuss the purpose, scope and logistics of the group. Community connectors link others in their local community with activities and organisations that can help improve their quality of life.

BRAG was established in January 2019 in partnership with the Public Programmes Team [32]. The group is community led with an emphasis on generating solutions and not feeling restricted by traditional formats of involvement. Funded through a strategic award made from the Wellcome Trust to the Public Programmes team, BRAG’s remit is to provide expertise and advice on inclusive practices; to involve diverse audiences in the development, delivery and dissemination of health research. This responds to the strategic priorities of the Public Programmes Team, operating across GM health research infrastructure (including the MBRC and the MCRF) of developing ‘community-led’ priorities, projects and approaches. To date (winter 2020), BRAG have

- Received leadership training, with a focus on involvement in health research and training on health research processes and structure within Greater Manchester
- Advised on five research projects and on a number of public engagement and communications projects, including the rebrand of the Public Programmes team (to Vocal) to support greater reach within communities
- Developed Top Tips for Researchers wishing to work with BRAG
- Co-authored blogs, with academics and members of the Public Programmes team [37] and co-presented at national events eg. Equality and Diversity in Science Symposium 2019
- Co-designed and co-delivered (with Public Programmes) training on Inclusive Research for GM based health researchers.
- Acted as co-applicants on several funding bids to NIHR and others
- Begun to form strategic research partnerships with GM research infrastructure (eg. Manchester Cancer Research Centre)

Several researchers who attended the sandpit have also continued their relationship with BRAG i.e. bringing both research projects to them, suggesting working with BRAG to colleagues and working with BRAG to design research projects.

## Conclusion

Overall the community sandpit event was a good starting point to engage grass roots community organisations with PPIE and health research. It raised awareness of the different ways in which communities can be engaged and provided practical learning on how to ‘level the playing field’ between community and research organisations, while at the same time enabling community representatives to network and also speak face to face with researchers. The opportunity for community representatives to network and speak directly with researchers was also important in making research more accessible. The event enabled relationships to be formed and trust to be built so that more long-term engagement and involvement can then take place. Recognising the social capital such as community assets, expertise and innovation (often with minimal resource) can provide a change for academic institutions to learn how to access wider audiences and provide further opportunities for partnerships across different sectors.

If running something similar in the future we would consider:

- Giving more time for pitch development in Day one
- Redesigning the voting process to ensure the projects funded more clearly reflected community organisations priorities
- Provide a more specific brief about the types of activities that would be funded as although some of the pitches were excellent ideas they didn’t always focus enough on health research

The sandpit addressed many of the concerns and challenges outlined in the introduction to this paper, in particular demonstrating how dignity and respect can be addressed through a community-led, asset-based and partnership approach. The sandpit has further demonstrated impact through its legacy in BRAG and chimes with recent research culture initiatives emphasizing the need for greater race equality and inclusion in research [25, 38].

## Supplementary Information

**Additional file 1.**

## Data Availability

Data sharing is not applicable to this article as no datasets were generated or analysed during the current study.

## Abbreviations

BAME: Black Asian Minority Ethnic
BRAG: Black Asian and Minority Ethnic Research Advisory Group
GM: Greater Manchester
GMBME: Greater Manchester Black Asian and Minority Ethnic Network
GMCVO: Greater Manchester Centre for Voluntary Organisations
GMPSTRC: Greater Manchester Patient Safety Translational Research Centre
MBRC: Manchester Biomedical Research Centre
MCRF: Manchester Clinical Research Facility
NIHR: National Institute of Health Research
PPIE: Patient and Public Involvement and Engagement
VCS: Voluntary and Community Sector
