# Supplementary material for: “We are not hard to reach, but we may find it hard to trust” …. Involving and engaging ‘seldom listened to’ community voices in clinical translational health research: a social innovation approach
Source: Res Involv Engagem. 2021 Jun 26;7:46. doi: 10.1186/s40900-021-00292-z (PMC8234650; doi:10.1186/s40900-021-00292-z)
Supplement: Supplementary file 1 — Additional file 1. [file 40900_2021_292_MOESM1_ESM.docx]

**Table three – Overview of Sandpit activities by day**

The sandpit event consisted of two days of workshops.

| **Day one – facilitated activities to enable community organization representatives (maximum of 10) to familiarize themselves with PPIE) within research across Greater Manchester and community artists who have experience of creatively engaging communities on health and well-being projects.**  **Tuesday July 17^th^, Manchester Central Library**  **Day 1. Community focus - levelling the playing field**  9.30am: Breakfast /Tea/Coffee - Registration  10.00am: Introductions, Rules of Engagement  10.30am: Creative activity - exploring research – “the unseen”.  11.30am: About engagement and involvement  12.30pm: Lunch  1.30pm: Role Play/storytelling: Exploring real case studies  2.45pm: Reflections and expectations for day 2 |
| --- |
| **Day 2 – Researchers join the discussions and highlight their research interests and how they want to work with diverse communities. After ice-breakers and networking activities the participants will work in small collaborative groups to devise a project to involve and engage their communities in research. These projects will then be pitched to the whole audience and after an interactive audience voting process five projects will be awarded funding (of £500-£750) at the end of day 2. It is hoped that researchers and practitioners will help facilitate the project development process and also be a collaborator on some of the projects.**  **Thursday 19^th^ July, Manchester Central Library**  **Day 2: Meet the researchers, Provocations, Innovation and Pitches**  9.30am: Breakfast/Tea/Coffee - Registration  10.00am: Introductions and overview of day  10.30am: Speaker tbc – Diverse Communities in Greater Manchester  11.00am: Overview of CVS sector – “We are not that hard to reach…”  11.30am: Speed meeting researchers/community organisations  12.30pm: Lunch  1.30pm: Come up with pitch (coffee break at own pace)  3.30pm: 3 minute pitch x 5-10  4.00pm: Voting  4.30pm announcing winners and next steps |
